# Respiratory Motion Compensation for Simultaneous PET/MR based on strongly undersampled radial MR data

**DOI:** 10.1186/2197-7364-2-S1-A24

**Published:** 2015-05-18

**Authors:** Christopher M Rank, Thorsten Heußer, Andreas Wetscherek, Marc Kachelrieß

**Affiliations:** German Cancer Research Center (DKFZ), Heidelberg, Germany

We propose a new method for PET/MR respiratory motion compensation, which is based on strongly undersampled measured MR data and a) runs in parallel with the PET acquisition, b) can be interlaced with clinical MR sequences, and c) can be acquired with measurement times as short as 0.5 min per bed position. An MR dataset covering the free-breathing thorax and abdomen of a volunteer was acquired with a Siemens Biograph mMR system. We applied a 3D encoded radial stack-of- stars sequence with a golden angle radial spacing (acquisition time: 5.0 min). Respiratory motion amplitudes were estimated from measured k-space centers allowing for a retrospective gating into 20 overlapping motion phase bins with a width of 10%. In addition, two highly undersampled datasets consisting of 300 and 600 spokes were created corresponding to acquisition times of 0.5 min and 1.0 min, respectively. 4D gated MR images of the three datasets (0.5, 1.0 and 5.0 min acquisition time) were reconstructed iteratively. For each of the three resulting image sets, MVFs were estimated. A 4D PET volume of the volunteer with four artificial hot lesions in the lungs and abdomen was simulated. 3D PET and MoCo 4D PET images based on the three sets of motion vector fields derived from MR were reconstructed and compared to a reference gated 4D reconstruction with ten-fold acquisition time. Visual inspection of the reconstructed PET images showed that blurring was reduced in MoCo 4D images for all acquisition times compared to the 3D reconstruction. A quantitative evaluation in the end-exhale and a mid-ventilation motion phase demonstrated that MoCo 4D reconstructions outperformed the 3D reconstruction in terms of SUVmean values for all lesions and acquisition times compared to the reference gated 4D reconstruction.

